# Overview of the Role of Vanillin on Redox Status and Cancer Development

**DOI:** 10.1155/2016/9734816

**Published:** 2016-12-19

**Authors:** Daniel Pereira Bezerra, Anne Karine Nascimento Soares, Damião Pergentino de Sousa

**Affiliations:** ^1^Gonçalo Moniz Institute, Oswaldo Cruz Foundation (IGM-FIOCRUZ-BA), Salvador, BA, Brazil; ^2^Department of Pharmaceutical Sciences, Universidade Federal da Paraíba, 58051-970 João Pessoa, PB, Brazil

## Abstract

Bioactive natural products play critical roles in modern drug development, especially anticancer agents. It has been widely reported that various pharmacological activities of such compounds are related to their antioxidant properties. Vanillin is a natural substance widely found in many plant species and often used in beverages, foods, cosmetics, and pharmaceutical products. Antioxidant and anticancer potential have been described for this compound. Considering the importance of vanillin in the area of human health and food and pharmaceuticals sectors, in this review, we discuss the role of vanillin on redox status and its potential contribution to the prevention and the treatment of cancer.

## 1. Introduction

A great body of in vivo evidence supports the view that oxidative stress and the accompanying reactive oxygen species (ROS) are genotoxic and contribute to the development of cancers. Pathological processes mediated by oxidative stress include cell membrane leakage, mitochondrial dysfunction, glutathione depletion, and disturbed redox cell state and depletion of ATP. These processes affect the cells and DNA, resulting in tumors, inflammatory diseases, and various other health problems. Oxidative stress is implicated in numerous diseases and results in shorter life, hampered well-being and increasing public health spending [1]. Antioxidant therapies implementation requires a better understanding of free radical toxicity, its molecular mechanisms, and its involvement in disease.

**Figure 1:**
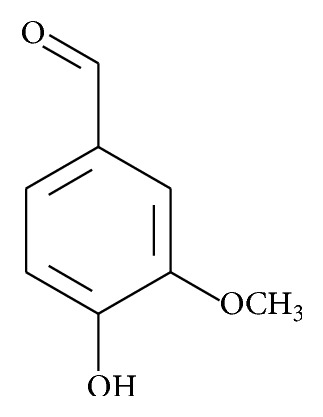
Chemical structure of vanillin (4-hydroxy-3-methoxybenzaldehyde).

Natural bioactive products play critical roles in anticancer drug development. In fact, natural molecules with antitumor activity reveal excellent pharmacotherapeutic potential [2–5]. Antioxidant activity is commonly found in many of these molecules, especially the phenolic substances [6, 7]. Considering the involvement of reactive species as a source of various types of cancer, diets and/or drug therapies involving bioactive substances with antioxidant activity may well represent a preventive treatment approach to maintain the well-being of the patient. Some of these natural substances are present in various pharmaceutical, nutritional, and cosmetic products. For example, vanillin (Figure 1), a plant secondary metabolite and the main constituent of vanilla, is a phenolic phenylpropane C_6_-C_1_ carbonic structure derivative. It acts as an important flavor and aromatic component used worldwide. Vanillin is found in several essential plant oils, principally* Vanilla planifolia*,* Vanilla tahitensis,* and* Vanilla pompona* [8]; it is often found in processed foods, beverages, and pharmaceutical products and also in perfumery [9, 10]. Vanillin has antioxidant and antitumor potential [11], and it is reported that its activity might be more beneficial for daily health care than had been previously thought [12]. A rich diet in such radical scavengers could reduce free radical cancer promotion [13]. Given the data on vanillin and considering its importance in its various applications and pharmacological properties, we discuss the role of vanillin on redox status and its potential contribution to cancer development and treatment.

## 2. Methodology

Searches were performed in the scientific literature databases: PubMed and Web of Science through June 2016 using the following key words: vanillin, antitumor, anticancer, oxidant and/or antioxidant.

## 3. Antioxidant and Prooxidant Effects

It is well known that neither O_2_^∙−^ nor H_2_O_2_ is capable of interacting with either deoxyribose or the base portions of DNA; this suggests that the secondarily derived ^∙^OH radical may be the primary reactive species. Once generated, ^∙^OH may interact with DNA to produce at least 20 different DNA oxidation products [14]. The idea that a major risk factor for cancer is mitogenesis has also been proposed. It has long been appreciated that dividing cells are at an increased risk for developing mutations as compared to quiescent cells [15]. Oxidants produced by inflammatory leukocytes may induce mutagenesis and possibly carcinogenesis by promoting mitogenesis and by modifying DNA bases [16]. There is an increasing interest in oxygen-containing free radicals in biological systems because of their perceived roles as causative agents in the etiology of a variety of chronic disorders. Many natural products having antioxidant activities show protective biochemical functions and such metabolites have become subjects of great interest. A better understanding of the mechanisms of oxidative biological reactions (and their inhibition) should help to understand and treat or prevent various diseases, such as cancer.

In regard of the antioxidant effect of vanillin, some mixed results have been reported. Vanillin had weak superoxide anion scavenging activity (IC_50_ of 2,945 ± 247 *μ*M) and exhibited no or little antioxidant activity against lipid peroxidation in mouse liver microsomes (inactive at 3,285 *μ*M) [17], 1,1-diphenyl-picrylhydrazyl (DPPH) radical scavenging assay (IC_50_ value higher than 100 *μ*M) [18], *β*-carotene decolorization assay [19], and linoleic acid and cholesterol oxidation assays [20]. On the other hand, vanillin at 2.5 mM inhibited protein oxidation and lipid peroxidation induced by photosensitization with methylene blue plus light in rat liver mitochondria [21]. It also exhibited hydroxyl radical [22] and 2,2′-azinobis (3-ethylbenzothiazoline-6-sulfonic acid) radical cation (ABTS^*∙*+^) (IC_50_ value of 16.25 *μ*M) [23] scavenging activities.

Furthermore, Tai et al. [12] assessed the antioxidant effect of vanillin using multiple antioxidant assays. Vanillin showed potent antioxidant activity in the ABTS^*∙*+^ scavenging assay, in the oxygen radical absorbance capacity (ORAC) assay, and in the oxidative hemolysis inhibition assay (OxHLIA), but showed no activity in the DPPH radical and galvinoxyl radical scavenging assays. Its antioxidant mechanism is via self-dimerization. The dimerization contributed to the high reaction stoichiometry against ABTS^*∙*+^ and 2,2′-azobis(2-methylpropionamidine) dihydrochloride- (AAPH-) derived radicals to result in the strong antioxidant effect of vanillin [12]. Pretreatment of vanillin (100 nM) also reduced rotenone induced mitochondrial dysfunction, oxidative stress, and apoptosis in SH-SY5Y neuroblastoma cells [24]. These data reinforce that vanillin presents interesting antioxidant properties, which is dependent on the method used.

In in vivo antioxidant assay, mice were orally given a single dose (100 mg/kg) of vanillin. The highest activity in the plasma ORAC assay was observed at 5 min, when the concentrations of vanillin, vanillic acid, and protocatechuic acid were high [12]. Moreover, pretreatment with vanillin (150 mg/kg) presented hepatoprotective effect against carbon tetrachloride-induced hepatotoxicity by the inhibition of protein and lipid oxidation processes, the increase in antioxidant enzymes activities, and the inhibition of inflammation mediators [25]. More recently, Ben Saad et al. [26] demonstrated the protective effects of vanillin against potassium bromate- (KBrO_3_-) induced oxidative stress. The treatment with vanillin (100 mg/kg/day for 15 days) reduced histopathological changes in KBrO_3_-induced kidney and decreased renal oxidative damage by inhibiting ROS generation and reversing antioxidant enzyme activities in kidney [26]. These effects may be results of interactions between vanillin and cellular proteins or signaling pathways.

In relation of the prooxidant effect of vanillin, Castor et al. [27] have reported that vanillin can has prooxidant action when its transient free radicals are generated intracellularly. Transient free radicals from vanillin were generated by horseradish peroxidase/hydrogen peroxide-mediated oxidation and their prooxidant effects were assessed using cysteine, glutathione, ovalbumin, and the coenzyme NADPH as target biomolecules. Vanillin, at concentration of 10 *μ*M, promoted oxidation of glutathione, sulfhydryl groups, and NADPH at experimental conditions [27]. This prooxidant activity can explain its cytotoxic action in some tumor cell lines. Briefly, we believe that both antioxidant and prooxidant characteristics of vanillin contribute to its benefits and deleterious effects. The antioxidant activity of vanillin seems to be related to its antimutagenic and anticarcinogenic activities. In contrast, the prooxidant effects of vanillin radicals can be related to its anti-invasive, antimetastatic, antiangiogenic, and cytotoxic activities.

The antioxidant activity of phenolic substances has been reported for several natural substances structurally similar to vanillin. The hydroxyl of the aromatic ring plays an important role in the antioxidant activity via the homolytic fragmentation of the O-H bond [28, 29]. It has been reported that vanillin exhibited hydroxyl radical [30] and 2,2′-azinobis (3-ethylbenzothiazoline-6-sulfonic acid) radical cation (ABTS^*∙*+^) [23] scavenging activities. Its antioxidant activity can be attributed to the presence of hydroxyl group (OH^*∙*^) linked to aromatic ring. Therefore, substances having the phenol functional group may also have antioxidant activity, as exhibited by vanillin.

## 4. Antimutagenic Activity

Mutagenic agents are involved in genotoxicity and carcinogenesis processes and as well in the inception and pathogenesis of several chronic degenerative diseases. Antimutagenic agents are the compounds that have protective effects against mutagenics, which include “desmutagens” that cause chemical and biochemical mutagen modifications before DNA damage and “bioantimutagens” that reduce the mutation process after DNA damage [31]. Vanillin has been classified as a bioantimutagen and is able to inhibit mutagenesis induced by chemical and physical mutagens in various cell systems.

The antimutagenic effect of vanillin was first tested on bacteria [32]. Vanillin (at concentration of 150 *μ*g/mL [~986 *μ*M]) inhibits mutagenesis induced by 4-nitroquinoline 1-oxide (4-NQO), furylfuramide (AF-2), captan, or methylglyoxal in* Escherichia coli* WP2s. In studies using repair enzymes in deficient strains, it was suggested that vanillin increased DNA damage repair through the recombinational repair pathway [32–34]. Vanillin was not effective against mutations provoked by 3-amino-l-methyl-5*H*-pyrido[4,3-*b*] indole (Trp-P-2) or 2-amino-3-methylimidazo[4,5-*f*]quinoline (IQ) in* Salmonella typhimurium *TA98 [32], yet it reduced the frequency of spontaneous mutations in* S. typhimurium* TA102 and TA104, reducing mutations at GC sites, but not AT sites, and such antimutagenic effect was dependent on the presence of the pKM101 plasmid in homologues of TA104 [35, 36]. Vanillin was also effective against spontaneous mutation in the wild-type strain NR9102 of* E. coli, *and this effect was independent of both the SOS and NER pathways. Moreover, vanillin causes certain types of DNA damage, which elicit recombinational repair. This recombinational repair activation permits vanillin-induced damage repair and also that of other DNA lesions, thus reducing the frequency of spontaneous mutation [37].

The antimutagenic effect of vanillin has also been studied in* Drosophila melanogaster*. In the germ cell line of* D. melanogaster*, vanillin decreased both spontaneous and mitomycin C- (MMC-) induced ring X-loss but had no effect on the mutagenic activity of methyl methanesulfonate (MMS) [38]. On the other hand, vanillin weakly inhibited mutations induced by MMC but increased recombination in MMC-treated lesions in* D. melanogaster* somatic cells [39]. Posttreatment with vanillin produced a synergistic effect on recombination in* D. melanogaster *somatic cells with either ethyl methanesulfonate (EMS) or bleomycin [40]. However, the effects of cotreatment with vanillin led to significant protection against the general genotoxicity of EMS,* N*-ethyl-*N*-nitrosourea (ENU),* N*-methyl-*N*-nitrosourea (MNU), and bleomycin [41]. Using the DNA repair test for* D. melanogaster*, [42] reported the effects of vanillin on the repair of lethal damage produced by* N*-ethyl-*N*-nitrosourea (ENU), ethyl methanesulfonate (EMS), and MMC. Vanillin also increased the toxicity of MMC and EMS in repair-deficient flies. Yet, it protected against the lethality of ENU in repair-defective flies. This complex antimutagenic effect has been attributed to vanillin caused inhibitory activity against oxidative damage and stimulatory action on detoxification enzymes [41, 42].

Contradictory results were also found in studies using mammalian cells. In studies performed in Chinese hamster ovary fibroblast CHO K-1 cells, vanillin was able to promote an increase in the frequencies of sister-chromatid exchanges (SCE) in* N*-ethyl-*N*′-nitro-*N*-nitrosguanidine- (ENNG-), MMC-, EMS-, ENU-, and MNU-treated cells, but not* N*-methyl-*N*-nitrosoguanidine- (MNNG-) or MMS-treated cells. The effect was S-phase-dependent in MMC-treated cells [43, 44]. In contrast, the frequency of chromosome aberrations was significantly decreased in MMC-treated cells in phase G_2_, by posttreatment with vanillin [44]. In addition, vanillin-treatment in the G_1_ phase suppressed X-ray-induced breakage-type and exchange-type chromosome aberrations. Treatment with vanillin in phase G_2_ suppressed ultraviolet light (UV) and X-ray-induced breakage types, but not exchange-type chromosome aberrations [45]. In experiments performed in Chinese hamster lung fibroblast V79 cells, vanillin reduced the frequencies of 6-thioguanine-resistant mutations induced by UV, X-ray, and ENU [46]. The cytotoxicity and chromosomal aberrations induced by hydrogen peroxide (H_2_O_2_) were suppressed when V79 cells were posttreated with vanillin. However, vanillin increased EMS-induced toxicity [47]. Vanillin also reduces methotrexate-, X-ray- and UV-induced micronucleated and binucleated aberrant cells [48, 49].

The in vivo antimutagenic effects of vanillin were studied in X-ray- and MMC-induced micronuclei in mouse bone marrow cells. Posttreatment with vanillin caused decreases in the frequency of X-ray- and MMC-induced micronucleated polychromatic erythrocytes [50, 51]. Vanillin was also investigated in the mouse spot test using male PW and female C57BL/10 mice. Three successive oral administrations of vanillin at 500 mg/kg decreased the ENU-induced frequency of recessive carrier pups [46].

The ability of vanillin to inhibit photosensitization-induced single-strand breaks (ssbs) in plasmid pBR322 DNA has been examined in a cell-free system, independent of DNA repair/replication processes. Vanillin was found to provide effective DNA protection against photosensitization-induced ssbs (mainly type II reaction) in the absence of DNA repair, replication machinery, or other cellular defenses, which can be in part due to its ability to scavenge O_2_ [23, 52]. On the other hand, vanillin was able to inhibit mutations at the* CD59* locus in human-hamster hybrid A_L_ cells induced by H_2_O_2_, MNNG, and MMC, but not ^137^Cs *γ*-radiation. The effects were attributed to the ability of vanillin to inhibit the DNA repair process that leads to the death of potential mutants or to enhancement of DNA repair pathways that protect against mutation but create lethal DNA lesions during the repair process [53]. In addition, vanillin is able to block DNA repair by nonhomologous DNA end-joining (NHEJ) and to selectively inhibit DNA-protein kinase (DNA-PK) activity in experiments using human lymphoma GM00558 cells and related gene deficient cells. Vanillin presented no detectable effects on other steps of the NHEJ process, on unrelated protein kinase, or on DNA mismatch repair in cell extracts. Vanillin also potentiated the cytotoxicity of cisplatin but did not affect sensitivity to UV [54].

The antimutagenic potential of vanillin was also assessed using both spontaneous and IQ-induced micronucleus frequencies in human hepatocellular carcinoma HepG2 cells. Vanillin caused a moderate increase in micronucleus numbers at the high concentration tested (500 *μ*g/mL [~3,286 *μ*M]); however, posttreatment of the cells with vanillin was able to inhibit IQ-induced micronuclei [55]. In addition, global gene expression in vanillin-treated mammalian cells was assessed against spontaneous mutagenesis in mismatch repair- (MMR-) deficient human colon cancer HCT116 cells. At concentrations of 0.5–2.5 mM, vanillin decreased the spontaneous mutant fraction. At concentrations (0.5–2.5 mM) that were antimutagenic in HCT116 cells, vanillin caused DNA damage in both mismatch repair-proficient (HCT116 + chr3) and deficient (HCT116) cells. A total of 64 genes presented the expression changed in vanillin- treated-HCT116 cells (2.5 mM for 4 h), including genes related to oxidative damage, stress response, DNA damage, apoptosis, and cell growth, demonstrating that DNA damage contributes to vanillin's antimutagenic effect [56]. Moreover, vanillin (100 *μ*M) reduced UV-induced cytotoxicity and DNA damage in human keratinocyte stem cells. It also increased the production of proinflammatory cytokines and decreased the phosphorylation of ataxia telangiectasia mutated (ATM), tumor suppressor protein 53 (p53), c-Jun N-terminal kinase/stress-activated protein kinase (JNK), serine threonine kinase checkpoint kinase 2 (Chk2), p38/mitogen-activated protein kinase (p38), S6 ribosomal protein (S6RP), and histone 2A family member X (H2A.X), suggesting that the ATM/p53 pathway is involved in the vanillin-induced mutagen protective mechanisms [57].

The protective effect of vanillin against KBrO_3_ induced liver, bone, and blood disorders has also been investigated [58, 59]. Coadministration of vanillin to KBrO_3_-treated mice significantly prevented DNA damage, hepatic cell alteration, and plasma transaminases increases, inhibited hepatic lipid peroxidation, and attenuated depletion of enzymatic and nonenzymatic antioxidants and expression levels of proinflammatory cytokines, including tumor necrosis factor-*α*, interleukin-1*β*, interleukin-6, and COX2.

**Figure 2:**
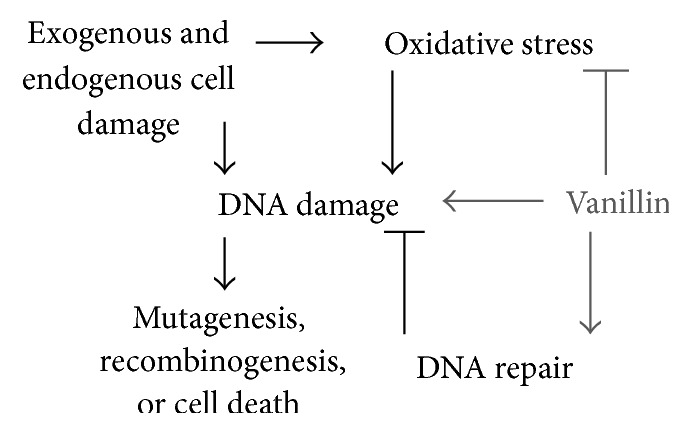
Overview of the antimutagenic effect of vanillin.

Although vanillin has shown comutagenic effects in some models, its antimutagenic effect (in concentration range in *μ*M) was extensively evaluated and appears to be due to its effects on cell redox and DNA repair pathways (Figure 2).

## 5. Anti-Carcinogenic Activity

Since vanillin presents antimutagenic effect in differing models and based on the close relationship between antimutagenic and anticarcinogenic activities, it was expected that vanillin presents anticarcinogenic effect; however, few studies thus performed to investigate its anticarcinogenic effects have shown only contradictory results.

The anticarcinogenic effect of vanillin was first evaluated in the initiation stage of hepatocarcinogenesis using an animal model. In this model, the rats were given a diet containing 1% vanillin for 8 days. On day 7, the animals received a single dose of the hepatocarcinogen, IQ, and 12 h afterwards, a two-third partial hepatectomy for initiation, and 2 weeks thereafter, they were placed on a promotion regimen comprising phenobarbital and a single dose of D-galactosamine. Within 11 weeks, antioxidant effect was assessed by comparing values for preneoplastic placental glutathione S-transferase positive (GST-P^+^) foci. Vanillin significantly decreased the number of GST-P^+^ foci/cm^2^, suggesting inhibitory effects on hepatocarcinogenesis initiation induced by the food carcinogen IQ [60].

Vanillin was also evaluated in a medium term multiorgan rat carcinogenesis model. Carcinogen-treated male F344 rats received vanillin in the diet at a dose of 1%. Vanillin was administered from 1 day before and throughout the carcinogen exposure period or after completion of the initiation regimen. All surviving animals were sacrificed at the end of week 36. In this model, vanillin only weakly inhibited small intestine and lung carcinogenesis but significantly increased colon carcinogenesis in the initiation phase and also enhanced the development of stomach and renal lesions in the promotion phase [61].

Recently, the anticarcinogen or cocarcinogen effects of vanillin were studied in azoxymethane- (AOM-) induced aberrant crypt foci- (ACF-) bearing rats. AOM-challenged rats were treated with vanillin orally and intraperitoneally at low (150 mg/kg) and high (300 mg/kg) doses, and then ACF density, multiplicity, distribution, and gene expression were observed. Vanillin consumed orally had no effect on ACF; however, when administered through intraperitoneal injection at higher concentration vanillin was cocarcinogenic, which could well increase ACF density and multiplicity. The expression of colorectal cancer biomarkers, protooncogenes (beta-catenin↑ and FOS↑), recombinational repair (XRCC2↑), mismatch repair (PMS2↑), cell cycle arrest (p21↑ and cyclin B↓), and tumor suppressor gene (tumor suppressor gene p53↑) were also affected by vanillin [62, 63]. The mixed anticarcinogenic/cocarcinogenic results found for vanillin suggest that other carcinogen models must be evaluated to better understand the role of vanillin on carcinogenesis.

## 6. Anti-Invasive, Antimetastatic, and Antiangiogenic Activities

In cancer invasions, the key events of metastasis and angiogenesis are considered to be therapeutic targets for cancer prevention and treatment, and interestingly, vanillin presents anti-invasive, antimetastatic, and antiangiogenic activities in differing models.

In vitro studies reveal that vanillin, in noncytotoxic concentrations, inhibits invasion and migration of mouse mammary adenocarcinoma 4T1 cells and inhibits the enzymatic activity of their metalloproteinase 9 (MMP-9) matrix secretions [64]. In addition, vanillin (also in noncytotoxic concentrations) reduces invasive capacity, suppresses 12-*O*-tetradecanoylphorbol-13-acetate- (TPA-) induced enzymatic activity of MMP-9, and decreases the mRNA level of MMP-9 induced in HepG2 cells, which occurs via downregulation of the NF-*κ*B signaling pathway [65]. In the range of noncytotoxic concentrations, vanillin also exhibits inhibitory effects on hepatocyte growth factor- (HGF-) induced migration of human lung A549 carcinoma cells, this due to inhibitory activity of phosphatidylinositol 3-kinase (PI3K). The effect has no correlation with the antioxidant activity of vanillin [66].

The in vivo antitumor, antimetastatic, and antiangiogenic effects of vanillin have also been investigated. The effect of vanillin on the growth and metastasis of 4T1 cells was assessed in BALB/c mice. In this model, the 4T1 cells were injected into the mammary fat pad in female mice. Vanillin (100 mg/kg) was orally administrated 6 times per week for one month, starting on the first day after tumor implantation. Vanillin-treated animals showed significantly reduced numbers of metastasized lung colonies as compared to the controls, without any inhibitory effect on primary tumor growth [64]. The in vivo antiangiogenic activity of vanillin was determined through inhibition of minor blood vessel formation (in chick chorioallantoic membrane; CAM assay) between day 3 and day 4 of chick embryo development. After exposure to vanillin for 24 h, inhibition of the minor blood vessel formation in day 4 eggs in the areas under the agarose pellets was observed at dosage in the range 100–500 nmol/pellet egg [66]. Despite the promising results found for the anti-invasive, antimetastatic, and antiangiogenic activities of vanillin, the data are limited. Further studies must be performed in new models to better characterize the effects and molecular pathways of vanillin.

## 7. Cytotoxic Activity

Vanillin in high concentrations (mM range) has been described as a cytotoxic agent against many cell lines, including mouse fibroblast 3T3 cells [67, 68], human ovarian carcinoma A2780-SC1 cells [54], human colorectal carcinoma HT-29 cells [68], HepG2 cells [69], human cervical carcinoma HeLa cells [70], and human colorectal carcinoma SW480 cells [71]. However, only a few studies investigated vanillin's cytotoxic mechanism of action.

The vanillin-treatment gene expression profile was evaluated [69] in HepG2 cells. Genes downregulated by vanillin were grouped into three gene ontological categories: regulation of cellular process, cell cycle, and death. Further, most of the downregulated genes were associated with cancer progression. Analysis of Fos-related transcription factor activator protein 1 (AP-1) showed that vanillin inhibits AP-1 activity, while diminishing the phosphorylation of extracellular signal-regulated protein kinase (ERK), thus indicating vanillin-regulated AP-1 activity via the ERK pathway. In other results [68], vanillin was able to induce both cytolytic and cytostatic effects in HT-29 cells. It also led to cell death through apoptosis pathways. Cell cycle analysis showed that vanillin induces G_0_/G_1_ arrest at lower concentrations (200 *μ*g/mL [~1,315 *μ*M]), while leading to G_2_/M arrest at higher concentrations (1000 *μ*g/mL [~6,573 *μ*M]). In addition, pretreatment of HeLa cells with vanillin enhanced tumor necrosis factor-related apoptosis-inducing ligand (TRAIL) induced cell death through inhibition of NF-*κ*B activation [70]. As cited above, the cytotoxic effect of vanillin is yet poorly reported, but based on the fact that it presents cytotoxicity only in high concentrations, its potential as a cytotoxic agent is low. The therapeutic potential of vanillin seems to be linked to its chemopreventive, anti-invasive, antimetastatic, and antiangiogenic actions (Table 1).

## 8. Conclusion

The studies presented in this review reveal vanillin's antioxidant activity, its antitumor action, and its therapeutic potential in cancer treatment and prevention. Consequently, the use of vegetables rich in this natural product might well be useful in inhibiting the free radicals responsible for tumor development. The data reported in this review are in accordance with the scientific understanding that a better quality of life and increased longevity may be obtained via healthy food, with the beneficial action of natural bioactive products.

## Figures and Tables

**Table 1:** Summary of the main molecular targets of vanillin.

Biological process	Molecular target
Cell proliferation and cell cycle	Akt↓, AP-1↓, ATM↓, aurora kinase B↓, calcyclin↓, Chk2↓, CTGF, cyclin A2↓, cyclin B↓, cyclin D1↓, ERK↓, H2A.X↓, INSIG1↓, I*κ*B-*α*↓, JNK↓, JUND↓, MAP2K2↓, MARK4↓, MMP-9↓, NF-*κ*B↓, p38↓, p65↓, PCNA↓, PI3K↓, polo-like kinase 1↓, S6RP↓, survivin↓, TGFB1I1↓, *β*-catenin↑, FOS↑

DNA damage, oxidative, and stress responses	CLK2↑, DDIT4↑, GCLC↑, GCLM↑, HMOX1↑, HSPA1B↑, PMS2↑, SLC7A11↑, TRIM16↑, UGP2↑, XRCC2↑, DNA-PK↓, DNMT3B↓, DUSP6↓, FGFR2↓, FZD2↓, NHEJ↓, PPP1R10↓, SLC35D2↓, UNG2↓

Apoptosis	p21↑, p53↑

Invasion and metastasis	MMP-9↓, PI3K↓

Akt, protein kinase B; AP-1, activator protein 1; ATM, ataxia telangiectasia mutated; Chk2, serine threonine kinase checkpoint kinase 2; CLK2, CDC-like kinase 2; CTGF, connective tissue growth factor; DDIT4, damage-inducible transcript 4; DNA-PK, DNA-protein kinase; DNMT3B, DNA (cytosine-5-)-methyltransferase 3 beta; DUSP6, dual specificity phosphatase 6; ERK, extracellular signal-regulated protein kinase; FGFR2, fibroblast growth factor receptor 2; FZD2, frizzled homolog 2 (Drosophila); GCLC, glutamate-cysteine ligase, catalytic subunit; GCLM, glutamate-cysteine ligase, modifier subunit; H2A.X, histone 2A family member X; HMOX1, heme oxygenase (decycling) 1; HSPA1B, heat shock 70 kDa protein 1B; INSIG1, insulin induced gene 1; I*κ*B-*α*, nuclear factor of kappa light polypeptide gene enhancer in B-cells inhibitor, alpha; JNK, c-jun N-terminal kinase/stress-activated protein kinase; JUND, jun D proto-oncogene; MAP2K2, mitogen-activated protein kinase kinase 2; MARK4, MAP/microtubule affinity-regulating kinase 4; MMP-9, matrix metalloproteinase 9; NF-*κ*B, factor nuclear kappa B; NHEJ, non-homologous DNA end-joining; p21, cyclin-dependent kinase inhibitor 1; p38, p38/mitogen-activated protein kinase; p53, tumor suppressor protein 53; p65, transcription factor p65; PCNA, proliferating cell nuclear antigen; PI3K, phosphatidylinositol 3-kinase; PMS2, mismatch repair endonuclease; PPP1R10, protein phosphatase 1, regulatory subunit 10; S6RP, S6 ribosomal protein; SLC35D2, solute carrier family 35, member D2; SLC7A11, solute carrier family 7; TGFB1I1, transforming growth factor beta 1 induced transcript 1; TRIM16, tripartite motif-containing 16; UGP2, UDP-glucose pyrophosphorylase 2; UNG2, uracil-DNA glycosylase 2; XRCC2, DNA repair protein.

## Abbreviations

4-NQO: 4-Nitroquinoline 1-oxide
AAPH: 2,2′-Azobis(2-methylpropionamidine) dihydrochloride
ABTS^*∙*+^: 2,2′-Azinobis(3-ethylbenzothiazoline-6-sulfonic acid) radical cátion
ACF: Aberrant crypt foci
AF-2: Furylfuramide
AOM: Azoxymethane
AP-1: Activator protein 1
ATM: Ataxia telangiectasia mutated
Chk2: Serine threonine kinase checkpoint kinase 2
DNA-PK: DNA-protein kinase
DPPH: 1,1-Diphenyl-picrylhydrazyl
EMS: Ethyl methanesulfonate
ENNG: *N*-Ethyl-*N*′-nitro-*N*-nitrosguanidine
ENU: *N*-Ethyl-*N*-nitrosourea
ERK: Extracellular signal-regulated protein kinase
GST-P^+^: Preneoplastic glutathione S-transferase placental form-positive
H2A.X: Histone 2A family member X
H_2_O_2_: Hydrogen peroxide
HGF: Hepatocyte growth factor
IQ: 2-Amino-3-methylimidazo[4,5-*f*]quinoline
JNK: c-Jun N-terminal kinase/stress-activated protein kinase
MMC: Mitomycin C
MMP-9: Matrix metalloproteinase 9
MMS: Methyl methanesulfonate
MNNG: *N*-Methyl-*N*-nitrosoguanidine
MNU: *N*-Methyl-*N*-nitrosourea
NF-*κ*B: Factor nuclear kappa B
NHEJ: Nonhomologous DNA end-joining
ORAC: Oxygen radical absorbance capacity
OxHLIA: Oxidative hemolysis inhibition assay
p38: p38/mitogen-activated protein kinase
p53: Tumor suppressor protein 53
potassium bromate: KBrO_3_
PI3K: Phosphatidylinositol 3-kinase
S6RP: S6 ribosomal protein
SCE: Sister-chromatid exchanges
ssbs: Single-strand breaks
TPA: 12-*O*-Tetradecanoylphorbol-13-acetate
TRAIL: Tumor necrosis factor-related apoptosis-inducing ligand
Trp-P-2: 3-Amino-l-methyl-5*H*-pyrido[4,3-*b*]indole
UV: Ultraviolet light.
